# Outdoor bicycle training following stroke: protocol development, feasibility testing and application guidelines

**DOI:** 10.3389/fresc.2026.1661479

**Published:** 2026-03-05

**Authors:** Myriam Villeneuve, Julie Piché, Antoinette Busch, Anouk Lamontagne

**Affiliations:** 1 School of Physical and Occupational Therapy, McGill University, Montreal, Quebec, Canada; 2 Feil and Oberfeld Research Centre, Jewish Rehabilitation Hospital Site of CISSS Laval, Centre for Interdisciplinary Research in Rehabilitation of Greater Montreal, Laval, Quebec, Canada

**Keywords:** cerebrovascular accident, cycling, feasibility, leisure, protocol, rehabilitation

## Abstract

**Background:**

The benefits of cycling on health have been extensively shown and indoor cycling is increasingly used as physical activity in stroke rehabilitation. However, standardized protocols and guidelines for outdoor bicycle training are still lacking for this population.

**Aims:**

To develop a structured, group-based training protocol for outdoor cycling skills in individuals with post-stroke hemiparesis and to test the feasibility of the training protocol in individuals with stroke.

**Methods:**

Based on existing literature, guidelines for other populations and the team's expertise, a training protocol and progression criteria were generated.

**Participants:**

Subsequently, five stroke participants engaged in the three-week bicycle training program that involved nine sessions (three sessions/week). Feasibility was assessed by documenting adherence, progression, adverse events, acceptability of the intervention, as well as the limited efficacy of the intervention on participant's cycling goals [Goal Attainment Scale (GAS)], self-reported confidence in cycling skills (CCS), participation in leisure (Nottingham Leisure Questionnaire) and other balance and mobility outcomes.

**Results:**

A detailed intervention protocol initially comprising of three modules of increasing complexity and that was extended to four modules to accommodate participants’ rapid progression was developed. Participants completed two to four modules, showed high intervention adherence and acceptability, and minimal adverse events. Post-intervention, significant improvements in GAS (*p* = 0.009) and CCS (*p* = 0.015) were observed, but marginal or no differences were found for all other outcomes.

**Conclusions:**

This manuscript presents the first, openly available stroke-specific training protocol for outdoor cycling. Findings suggest that the intervention is feasible and acceptable, and that it improves outdoor cycling proficiency.

## 1 Introduction

Stroke is a worldwide health issue and one of the main causes of death and disability (1). In Canada, it is estimated that half of stroke survivors are left with disability that requires intensive rehabilitation, long-term assistance for daily activities and support in the community (2). As populations live to older ages, stroke prevalence and the long-term deficits associated with this chronic condition are also expected to increase considerably (3). These long-term deficits result in limited participation, mostly regarding mobility outside the home and participation in leisure and community activities (4). Restrictions in participation is a major concern post-stroke as it is fundamental to an individual's well-being and life satisfaction (5, 6). Participation in physical activity following stroke was shown to improve independence in activity of daily living, general health, and quality of life (7, 8). Despite these known benefits, however, most individuals with stroke, including those who sustained a mild stroke, do not return to pre-injury, high-demand leisure activities that involves physical activity (9), ultimately leading to an engagement in more ‘passive’ activities and sedentary lifestyle (10, 11).

The physical benefits of cycling as a physical activity have been well researched. In the literature, stationary cycling is one of the predominant exercises used among stroke survivors to improve aerobic capacity (12, 13) as well as motor control and muscle strength in the paretic lower extremity (14, 15). Cycling outdoors, however, differs in many ways from stationary cycling, not only in terms of sensorimotor and cognitive demands, but also as a multifaceted leisure activity and a mode of transportation (16, 17). In Canada, where the study presented in this manuscript was conducted, outdoor cycling is the second most popular outdoor leisure activity (18), with approximately 40% of the Canadian population aged 12 or older riding a bicycle at least once a year (19). In some European countries such as the Netherlands, where there are 1.3 times more bicycle than people, 28% of the population use a bicycle as its primary mode of transportation (20). In that same country, older adults aged between 65 and 74 years cycled on average 24 km per week in 2020 (21). Beyond the positive effects of exercise on mood and well-being (22, 23), the open environment, fresh air and scenic views associated with outdoor cycling offer a more dynamic and engaging experience and were suggested to be factor that also contributes to the well-being of older adults practicing outdoor cycling (24). It was also shown that more time spent outdoor at daytime is associated with better mood and sleep (25). Furthermore, outdoor cycling presents the advantage of continuously challenging balance by negotiating multiple and changing environmental factors (such as terrain variation, wind resistance and road condition), requiring increased efficiency and adaptability in terms of postural adjustments compared to stationary cycling. In fact, compared to non-cyclists, outdoor healthy adult cyclists have been shown to display enhanced postural strategies, leading to higher stability (26). Cycling outdoor could therefore be beneficial to improve balance following stroke. Another significant benefit of cycling outdoor is the active engagement in a leisure activity, which was shown to contribute to increased psychological well-being, including quality of life and mood in stroke survivors (27). Cycling within a group, when possible, could also increase social interactions as well as community participation, which is associated with reduced depression and isolation (28, 29). Group intervention is also an opportunity to learn from others. In fact, evidence show that the mirror neuron system can be activated in response to observation of actions performed by other people and therefore promote motor function recovery (30). Watching and imitating someone performing a cycling skill could therefore potentially stimulate one's learning abilities. Altogether, these finding advocate for group cycling outdoor as a mean to promote physical and mental health, both of which are affected following stroke (31). Such activity further has the potential to be sustained over the long term, thereby maximizing the emergence and maintenance of health benefits.

Currently, existing protocols and guidelines for outdoor bicycle training target healthy adult populations (32, 33). However, there is a major gap in the literature regarding such interventions for individuals with stroke. Meanwhile, interventions geared at improving cycling proficiency were successfully applied to various populations such as typically-developing children (34) and children with intellectual disability (35), as well as healthy adults primarily aged between 25 and 74 years (16). Individuals with stroke, however, present unique challenges due to persistent sensorimotor, cognitive, and perceptual impairments. These deficits notably impact posture, balance and movement coordination, as well as the ability to respond to environmental demands (e.g., obstacles, curb, etc.) in a timely fashion, all of which are essential for safe outdoor cycling (36). As such, these impairments not only limit the ability to return to cycling but also represent key rehabilitation targets that outdoor cycling could help address. Engaging in a structured outdoor cycling program may therefore provide a task-specific and ecologically-valid rehabilitation context to improve post-stroke impairments, while also facilitating reintegration into leisure and community activities. Individuals with stroke would therefore need a tailored program with even greater emphasis on safety precautions, given the consequences of falls on participation, quality of life and even mortality (37, 38). Moreover, unlike children learning the basic skills of cycling for the first time, stroke survivors build on previously acquired skills and must adapt their strategies to their current level of ability. Given these specific challenges, there is a need for a real-world outdoor cycling intervention that is grounded in established rehabilitation principles and tailored to the unique needs of individuals following stroke. Such an intervention should be informed by recognized principles of motor learning, including repetition, task specificity, progression of difficulty, feedback and saliency, all of which have been shown to support neuroplasticity and motor recovery (39). It should also align with the International Classification of Functioning, Disability and Health (ICF) by targeting not only impairments in body functions (e.g., balance, motor control), but also limitations in activity performance (e.g., cycling abilities) and participation (e.g., community mobility and leisure involvement). The present study was therefore designed to develop a structured, stroke-specific outdoor cycling protocol that integrates these theoretical foundations to promote both functional recovery and meaningful engagement in everyday life.

The current study also emerged from a request from rehabilitation therapists at our clinical site who, in the absence of a standardized training protocol and guidelines, were unsure of how to effectively and safely address the spontaneous demands of individuals with stroke to get support to return to outdoor cycling. The primary objective of this study was thus to develop and refine a group-based training protocol aimed at enhancing outdoor cycling skills in individuals with post-stroke hemiparesis. The secondary objective was to test the feasibility of the protocol in terms of its acceptability and the limited efficacy of the intervention on participant's personal cycling goals, self-reported confidence in cycling skills, balance, mobility, and participation in leisure activities. The constructs of acceptability and limited efficacy were identified as key areas to determine whether the intervention is worth testing in a future efficacy study (40).

## 2 Materials and methods

### 2.1 General procedures

#### 2.1.1 Protocol development (phase 1)

In order to develop the current cycling intervention protocol, existing protocols and guidelines designed for healthy children (34) and adults (32, 33), as well as a checklist adapted from a study conducted on children with intellectual disabilities (41) were extensively reviewed and relevant principles to an adult stroke population were identified and adapted. In addition, principles from the ergonomic guidelines established by Ergotec (42) were incorporated in order to maximize comfort and safety during training. The resulting intervention protocol was designed to allow individuals with stroke to train, as part of a group supervised by therapists, on functional cycling tasks that were progressively increasing in complexity. The initial protocol consisted of three modules of increasing difficulty. Within each module, a checklist of various skills required for safe and efficient cycling was included. This protocol was refined and completed with additional modules during Phase 2 of the study, based on real-time field experience with the participants.

#### 2.1.2 Feasibility testing (phase 2)

This second phase involved the feasibility testing of the intervention within the clinical settings. This phase of the study involved a multiple pre, multiple post study design. The intervention itself took place in the parking lot and surrounding neighborhood of the CISSS Laval Jewish Rehabilitation Hospital, a rehabilitation centre located in the greater Montreal area in Quebec, Canada. The project was approved by the Research Ethics Board *en réadaptation et en déficience physique du CCSMTL*. Each participant signed a consent form prior to their involvement in the study.

### 2.2 Participants

A convenience sample of five stroke survivors with mild-to-moderate motor impairments was recruited amongst discharged patients of the rehabilitation centre who had expressed the desire to return to cycling to their treating therapist during their rehabilitation.

As a feasibility study with a substantial risk of falls due to the nature of the intervention, the eligibility criteria for this study were conservative and determined so as to include participants who were most likely to be physically and cognitively capable to safely participate in the bicycle training program. Considering participant availability, personal interest in outdoor cycling, and strict eligibility criteria, the small sample size was nonetheless deemed acceptable for feasibility testing (43). Inclusion criteria were as follows: (1) having a hemorrhagic or ischemic supratentorial stroke; (2) being discharged from rehabilitation for less than five years, allowing for the attainment of steady-state mobility while also minimizing the risk of long-term disuse-related change; (3) being over the age of 18; (4) having ridden a bicycle in the two years preceding the stroke and; (5) considering outdoor cycling a leisure activity. In addition, participants had to present (6) a normal or corrected-to-normal visual acuity [score of 20/20 on the Early Treatment Diabetic Retinopathy Study (ETDRS)]; (7) mild-to-moderate motor impairments (score between 3 and 6 on the Chedoke-McMaster Stroke Assessment Impairment Inventory for either the arm or hand, as well as for either the leg or foot) (44); (8) a limited risk of falling (score of >45/56 on the Berg Balance Scale (45) and score of <14 s on the Timed Up & Go (TUG)) (46) as well as; (9) normal cognitive function or no more than mild cognitive impairments (score of >23/30 on the Montreal Cognitive Assessment) (47). Individuals with comorbidities interfering with cycling (e.g., orthopedic, rheumatological or neurological conditions other than stroke, comprehension aphasia, visuospatial neglect or field defect), or without medical clearance for exercise, were excluded.

### 2.3 Intervention

The initial bicycle training program developed under Phase 1 comprised of three modules of increasing complexity. Given the rapid and higher than expected progression of some participants, and since feasibility studies provide the opportunity to adapt an intervention as necessary and improve the procedures along the course of the study to achieve the most promising approach to a successful intervention (48), the training program was expanded to four modules. A summary of the training program and modules can be found in Table 1.

**Table 1:** Overview of bicycle training program.

Module 1: Stationary Bicycle
Mounting the stationary bicycle
Initiating cycling
Cycling continuously with steady rhythm
Shoulder checking
Dismounting the stationary bike
Module 2: Bicycle with training wheels
Walking with bicycle
Getting on and off bicycle
Initiating pedaling and stopping without help maintaining balance
Straight line riding
Stop quickly with control
Making sharp turns
Making U-turns
Steering to avoid obstacles
Signaling
Shoulder checking
Cycling on incline/decline
Module 3: Basic bicycle without training wheels
Walking with bicycle
Getting on and off bicycle
Initiating pedaling and stopping without help maintaining balance
Straight line riding
Stop quickly with control
Making sharp turns
Making U-turns
Steering to avoid obstacles
Signaling
Shoulder checking
Cycling on incline/decline
Module 4: Advanced bicycle skills
Vary speed
Practice loss of balance
Stall and resume biking
Passing another cyclist and being passed
Ride over obstacles
Drops: Ride down/off a curb
Narrow biking
Biking while standing
Biking single hand
Single leg pedaling
Narrow slalom
Narrow 360 degree turns when surrounded by obstacles
Predictable obstacle course
Unpredictable obstacle course
Application of all skills learned, in an uncontrolled setting

The intervention consisted of a structured three-week bicycle training program delivered in a group format, comprising nine sessions (three sessions per week), each lasting between 1.5 and 2 h. A three-week period was deemed optimal given the availability of clinicians and of the targeted population, who had terminated their rehabilitation and had reintegrated the community. Sessions were supervised by trained instructors with a 1:1 instructor-to-participant ratio for Modules 1 and 2, and a 2:1 ratio for Modules 3 and 4. This ratio allowed the instructor(s) to remain near the participant to provide physical assistance and assist with balance when needed, as well as to provide verbal and visual cues and feedback. All sessions took place outdoors on the premises of the rehabilitation hospital and nearby neighborhood streets, in environments selected to ensure safety while providing progressively increasing challenges. An ergonomic assessment was completed prior to the intervention to ensure that bicycles and helmets were appropriately adjusted to maximize safety and comfort (49). A printable version of a checklist, referred to as ‘Outdoor Cycling Safety Checklist’ can be found in Supplementary Appendix A.

The training program was divided into four modules, each designed to progressively build on cycling proficiency: The first module focused on stationary cycling tasks to reintroduce pedaling rhythm, motor control, and seated balance; the second module introduced functional cycling tasks using bicycles with training wheels in a controlled outdoor environment, emphasizing mounting, dismounting, braking, turning, and obstacle avoidance; the third module transitioned participants to standard bicycles without training wheels, with a focus on developing core competencies for safe, independent cycling; the fourth module, added during Phase 2 based on participant progress, introduced advanced cycling skills such as narrow turns, obstacle navigation, balance recovery, and integration in uncontrolled environments (e.g., road edges, curbs, and mixed terrain).

Each module comprised multiple levels that reflect increasing task complexity within the module. Participants progressed from one module to the next only after they had successfully demonstrated all skills listed for their current module on two consecutive trials, as determined by the instructor using a standardized “Skills Achievement Checklist” (see Supplementary Appendix B).

Safety precautions included constant instructor supervision, the use of walking belts in Modules 2 and 3, environmental control (e.g., use of cones to delineate areas), and staged exposure to more unpredictable environments. In the context of this feasibility study, a total of 4 instructors were involved, all of whom were in the terminal year of their degree in physical therapy or occupational therapy. They were supervised by AL and JP.

### 2.4 Evaluation of participants

**Figure 1:**
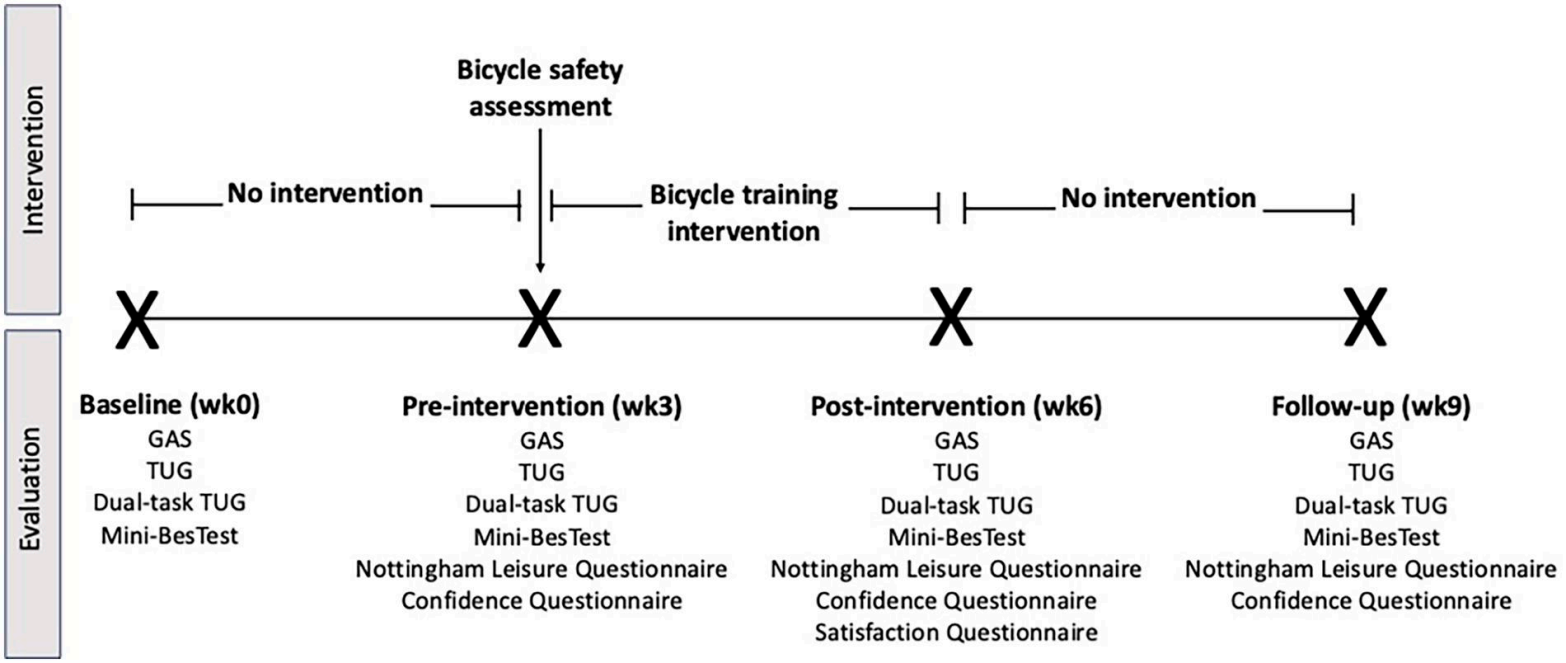
Study procedure and data collection timeline.

Participants underwent an evaluation at baseline (week 0), pre-intervention (week 3), post-intervention (week 6) and at follow-up (week 9). See Figure 1 for details on data collection timeline.

Acceptability, which measures the extent to which the new intervention is suitable and perceived as satisfactory to participants, was assessed through the following measures: (1) adherence to intervention (number of sessions and modules completed), (2) presence of adverse or undesirable events (i.e., falls, fatigue) collected though observation and open-ended questions, and (3) participant's perceived level of satisfaction with the intervention using a questionnaire developed by our research team and based on studied guiding questions (48). The latter questionnaire, administered post-intervention, included 10 questions that addressed participants’ satisfaction with the intervention in general, the demonstration and practice procedures, the timing and frequency of the training sessions, the participant to therapist ratio, and the level of challenge of proposed tasks (See Table 2). In response to the questions, participants either used a 5-item Likert scale to express their level of satisfaction, or answered “Yes” or “No”. This was complemented by open-ended questions about (i) elements participants enjoyed the most about the training program and (ii) recommendations for improving the quality of the intervention and (iii) participant's intention to pursue outdoor cycling as an activity following the intervention.

**Table 2:** Questions from the satisfaction questionnaire.

	Very Satisfied	Satisfied	Little satisfied	Dissatisfied	Very dissatisfied
How satisfied are you with the bike intervention?					
How satisfied were you with the demonstrations throughout the intervention?					
How satisfied were you with the explanations given throughout the intervention?					
Are you satisfied with the time allocated for practice throughout the intervention?					
How satisfied are you with the frequency of scheduled meetings throughout the intervention?					
How satisfied are you with the length of the various meetings allocated to the intervention?					
	Yes		No		
Do you think the time required (duration and number of meetings) for the intervention was realistic?					
Do you think the intervention was the right challenge for you?					
How satisfied are you with the ratio of participants to intervention managers?					
How likely would you be to recommend this procedure to another stroke patient wishing to return to cycling?					

Limited efficacy of an intervention assesses whether this intervention show promise of being successful with a specific population (40). In this study, the primary outcome to address limited efficacy pertained to participants’ goal achievement in terms of cycling, which was assessed using the Goal Attainment Scale (GAS). The GAS, which reflects the extent of goal achievement with scores ranging from −2 (nowhere near achieving the goal) to +2 (greatly surpassed the goal), was shown to be valid and to demonstrate excellent test-retest reliability in a geriatric population (50). As per the administration guidelines, a score of −1 was attributed to participants prior to the intervention (51). In addition, self-reported confidence in cycling skills was assessed using a scale specifically designed for that purpose by Telfer et al. (2006), which comprises of 15 elements pertaining to skills such as balancing on the bike, managing gears cycling uphill, managing road hazards, etc. (16). The scale was shown to be sensitive to change in an adult population engaged in a cycling proficiency program (16). Each item on this scale was assessed using a 5-item Likert scale (5 = very confident; 4 = quite confident; 3 = unsure; 2 = a little bit confident and 1 = not confident), yielding a maximal score of 75. Changes in balance were measured using the Mini-BESTest, while those in mobility were assessed using the Timed Up and Go (TUG), and dual-task TUG also referred to as the CogTUG. Both the Mini-BESTest and the TUG are valid and show excellent test-retest reliability with the stroke population (52, 53). The CogTUG was also shown to be valid and reliable when used with older adults (54). Finally, frequency of participation in leisure activities was assessed, as measured by the Nottingham Leisure Questionnaire (36-item version). The latter was found to have acceptable test-retest reliability (36). With the exception of the Self-Reported Confidence in Cycling Skills Questionnaire and Nottingham Leisure Questionnaire, which were not assessed at week 0, limited efficacy outcomes were assessed at all evaluation time points (Figure 1).

### 2.5 Statistical analysis

Results pertaining to acceptability and the Nottingham Leisure Questionnaire were collated and analyzed descriptively. As for outcome measures related to limited efficacy, they were compared across the different time points using non-parametric statistics. More specifically, outcome measures were first contrasted between the two baseline time points (week 0 and week 3) using a Wilcoxon signed rank test to ensure that participants were stable before commencing the bicycle training program. Since no significant differences were observed between the baseline and pre-intervention time points, the limited-efficacy analyses were conducted using Friedman tests that included outcomes measures collected immediately prior to the intervention (week 3), post-intervention (week 6), and at follow-up week 9). When statistically significant, the Friedman test was followed by *post-hoc* comparisons completed using Wilcoxon signed rank test with Bonferroni adjustments. The level of significance was set at *p* < 0.05 and all statistical analyses were conducted in SPSS.

## 3 Results

A standardized cycling training protocol was successfully developed by our research team and further refined during the second phase of the study by adding a fourth module to address more complex tasks requiring additional training from the participants. The detailed printable version of the program is presented in Supplementary Appendix B. Characteristics of participants who were included in the study are presented in Table 3. The sample consisted of individuals aged between 25 and 76 years (mean age of 63 years), predominantly males (*n* = 4), who had a stroke 4–43 months previously which, in the majority, took place in the right hemisphere (*n* = 4). Chedoke-McMaster Stroke Assessment scores ranged between 3 and 6 for the arm and hand, and between 3 and 7 for the leg and foot, indicating a wide range of severity of motor deficits. Berg balance scores at study enrollment varied between 49 and 56 (average of 52), reflecting moderate to high balance abilities (45, 55). Out of the five participants, only two had attempted bicycling after their stroke, one of them using a recumbent bike.

**Table 3:** Participant demographics.

Participant	Age	Gender	Time since stroke	Stroke		TUG score	Berg score	Chedoke-McMaster Stroke Assessment				
				Location	Etiology			Shoulder Pain	Arm	Hand	Leg	Foot
1	75	M	8	Left Sylvian	Ischemic	10	55	5	5	3	6	5
2	76	M	14	Right Fronto-parietal	Ischemic	9.38	55	5	5	5	6	3
3	68	M	43	Right Subcortical	Ischemic	13.97	49	N/A	5	5	6	4
4	25	F	4	Right Sylvian	Hemorrhagic	6.5	56	5	6	4	6	6
5	71	M	10	Right Lacunar	Ischemic	14	49	6	6	5	7	6
Average	63	4:1	15.8	-	-	10.77	52.8	5.25	5.2	4.6	6	5.2

Age (years), Gender (Male/Female), Time since stroke (months), TUG score (sec), Berg score (/56) Chedoke-McMaster Stroke Assessment scores (max=7); N/A: not available.

### 3.1 Adherence and progression

All participants completed the nine sessions, except one participant who missed one session due to personal constraints. Four participants attained all the competencies of Module 3 within the first 2 weeks of the training protocol and were able to progress through Module 4 at week 3. Participant #1 plateaued at Module 2 and was unable to attempt Module 3 due to an increased risk of falls identified by the intervention team. Adverse events included two falls in the early stages of the training program which occurred as participants were attempting to dismount their bicycle while stationary. These falls led to minor skin abrasions that were immediately taken care of but did not otherwise prevent the participation to the current training session. No participant reported fatigue during the training sessions.

### 3.2 Acceptability

Results from the satisfaction questionnaire show that all five participants gave the highest satisfaction rating (very satisfied) for every element pertaining to the intervention, including teaching and practice procedures, the module's progression, the participant to therapist ratio, and other elements further listed in Table 2. In addition, all participants reported that the time required (duration and number of sessions) for the intervention was realistic and that the intervention was the right challenge for them. When questioned about elements they enjoyed from the program, three participants reported that the increased confidence in their skills as well as encouragements from others were important factors contributing to their appreciation of the training. Two participants further mentioned that they also felt encouraged and motivated while watching other group members attempting different skills and that it helped them attempting skills that they might have never tried on their own. Other elements that were appreciated by at least one participant were the instructor's high involvement and connection during the practice sessions, the repetition/high intensity of practice, the smooth progression of the program and the opportunity to practice different biking skills. No recommendations to improve the quality of the intervention were proposed by participants. In terms of their intent to pursue the outdoor biking activity following the intervention, all five participants reported that they intended to bike regularly. At follow-up, four of them reported they had biked at least once between the post-intervention and follow-up evaluations.

### 3.3 Effects on outcomes related to cycling

**Figure 2:**
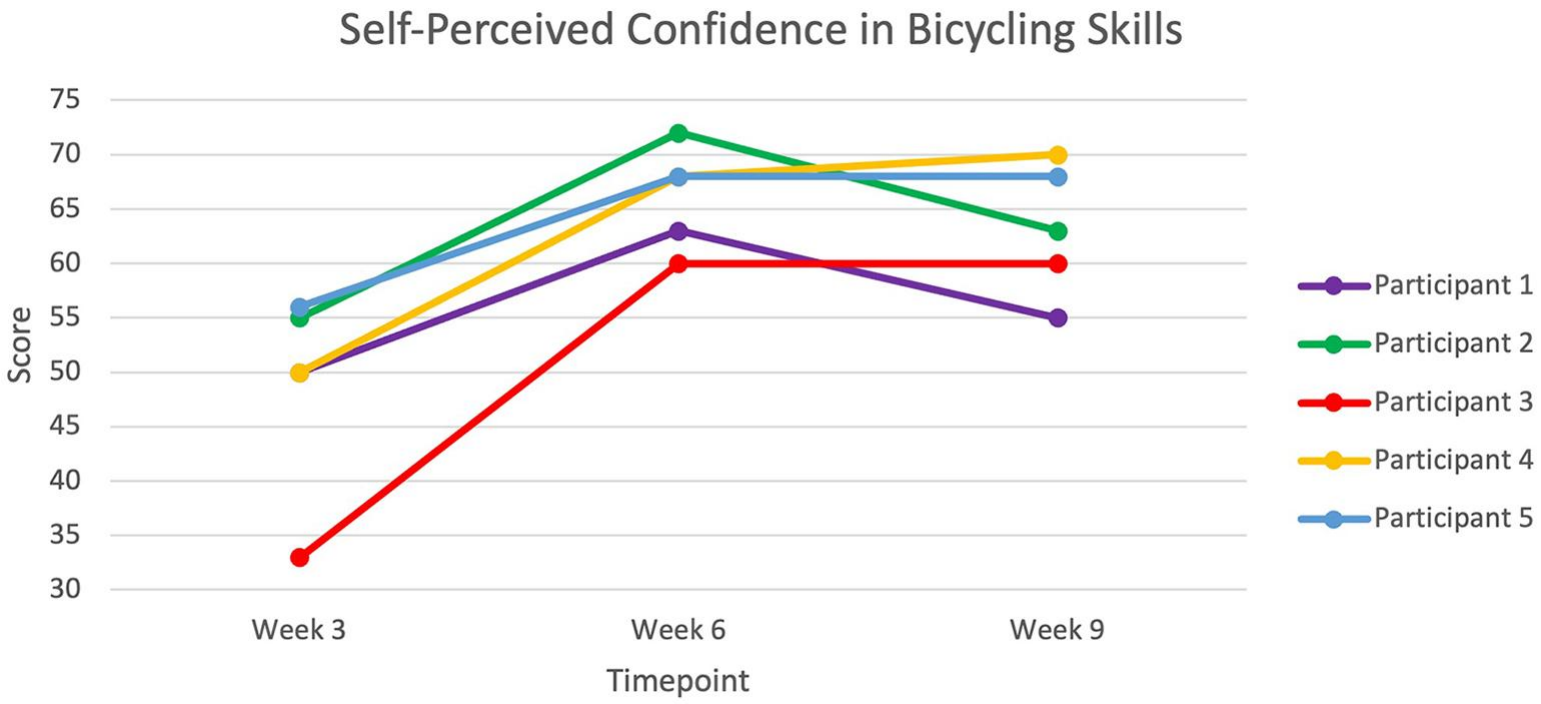
Individual performance for all participants. Maximal score = 75.

Results from the Friedman test showed significant differences across the different time points for the GAS (*p* = 0.009) and the Self-Reported Confidence in Bicycling Skills Questionnaire (*p* = 0.015). *Post-hoc* analyses revealed that results on both tests were significantly improved at post-intervention and follow-up compared to the pre-intervention levels (*p*-value range: 0.027–0.031), while showing no differences between post-intervention and follow-up measurement time points (*p*-value range: 0.564–0.317). Further examination of the GAS results (Table 4) showed that 4 participants either greatly surpassed (*n* = 2) or surpassed (*n* = 2) their goal, while a fifth participant (#1) partially achieved their goal and still needed supervision by the end of the Intervention. At follow-up, the achievement level was stable for all participants, except for participant #4 who's score went from “surpassed” to “greatly surpassed”. Of note, participant #2 who used a recumbent bike prior to the intervention was able to use a 2-wheeled bike during the training and participant #5 was able to bike for 60 km at the end of the intervention. As for the Self-Reported Confidence in Bicycling Skills Questionnaire (Figure 2), the increase in confidence was observed in all participants, with scores increasing by 21%–82% from pre- to post-intervention and 10%–82% at follow-up.

**Table 4:** Goal attainment scale: goals set by each participant and levels of achievement post-intervention and at follow-up.

Participant	Goal	Achievement level post-intervention (week 6)	Achievement level at follow-up (week 9)
1	Will be able to bike outdoor independently on a 4-wheel bike by the end of the intervention.	Score: **0**	Score: **0**
		Justification: Participant biked outdoor on a 4-wheels bike with supervision.	Justification: Participant biked outdoor on a 4-wheels bike with supervision.
2	Will be able to bike outdoor independently on a recumbent bike for 1 h by the end of the intervention.	Score: **+2**	Score: **+2**
		Justification: Participant biked on a 2-wheel bike independently for 10 km.	Justification: Participant biked on a 2-wheel bike independently for 10 km.
3	Will be able to bike outdoor independently for 20 min by the end of the intervention.	Score: **+2**	Score: **+2**
		Justification: Participant biked outside on different surfaces for 1 h.	Justification: Participant biked outside on different surfaces for 1 h.
4	Will be able to bike outdoor independently for 30 min, safely and without loss of balance by the end of the intervention.	Score: **+1**	Score: **+2**
		Justification: Participant biked in the city independently for 45 min.	Justification: Participant biked in the city independently for 2.5 h.
5	Will be able to ride a bike outdoor, keep your balance and react quickly to avoid obstacles by the end of the intervention.	Score: +**1**:	Score: +**1**:
		Justification: Participant biked 60 km outside.	Justification: Participant biked 60 km outside.

Scores for the Goal Attainment Scale go as follows: whether this goal was achieved as expected (0); if it was slightly exceeded (+1) or greatly exceeded (+2); or if it was partially achieved (−1) or nowhere near (−2).

### 3.4 Effects on balance, mobility and participation

Results from the Friedman test showed significant differences across the different time points for the CogTUG (*p* = 0.022), but not for the TUG (*p* = 0.165) and the Mini-BESTest (*p* = 0.779). *Post-hoc* analyses showed that results on the CogTUG were significantly improved both post-intervention (*p* = 0.031) and at follow-up (*p* = 0.031) compared to pre-intervention levels, with no significant difference between post-intervention and follow-up (*p* = 0.406). In response to the Nottingham Leisure Questionnaire, results from the Friedman test showed no significant difference across time points [*p* = 0.8 (32)]. A closer look at individual items of the questionnaire revealed that three participants (# 1,3,4) reported that their degree of participation in sport activities changed from never to occasionally after the intervention, whereas the two other participants’ level of participation stayed unchanged (#2 and 5). Please note that the latter two participants were already engaging in sport activities occasionally and regularly, respectively, prior to the intervention.

## 4 Discussion

The primary purpose of this study was to develop a standardized cycling group intervention protocol for individuals who had a stroke, and to make this protocol available to the clinical community and researchers. The protocol developed includes a detailed description of the tasks to be accomplished, which are presented in a logically progressive order, along criteria for progression. This protocol, developed in response to the needs of both rehabilitation therapists and their patients with stroke, proposes a short-duration, intensive group intervention tailored to the reality of the rehabilitation hospital with an outpatient stroke program. It can be easily adapted in terms of format, frequency, and duration to suit patient needs, endurance levels, and available treatment time.

This study additionally examined the feasibility of the bicycling intervention. Overall, results support its feasibility, as demonstrated by the excellent adherence, whereby participants attended every single session, with the exception of one participant who missed one session due to unexpected circumstances. Feasibility is further supported by the high acceptability of the intervention by all participants who gave the highest satisfaction ratings to all elements of the intervention they were questioned on, including elements such as training procedures, progression, patient/therapist ratio, frequency, intensity and length of the intervention. The intervention was also perceived as providing the right challenge to participants, which aligns with the fine progression of participants through various tasks of increasing complexity during the intervention. In fact, given that some of the participants progressed through the modules at a rate that was faster than anticipated, and given the need to address more complex cycling skills, a fourth module was created and tested.

Results also show that by the end of the intervention, four out of the five participants biked outdoor independently in a safe and effective manner and would not require supervision, which further support the feasibility of the intervention. The progression noted during the intervention was further corroborated by significant improvements in self-perceived confidence in bicycling skills and the fact that four out of five participants exceeded their personal cycling goal post intervention. Importantly, gains in terms of confidence and goal achievement were maintained at follow-up. Current literature on motor recovery after stroke supports the importance of developing training approaches that provides sufficient repetition to induce training effects, while being meaningful, challenging and tailored to the individual's capacity (56, 57), and we believe these elements, which were also highlighted in the participants’ feedback, were key to the success of the intervention. The group setting was another potential contributing element as it was brought up as a positive aspect of the intervention by most participants, especially when it comes to receiving encouragement from other participants and observing others which was considered inspiring and uplifting. While group interventions have been suggested to improve adherence and motivation, consistent evidence supporting this assertion, as well as the superiority of group vs. individual interventions in terms of health outcome improvements following stroke, has yet to be demonstrated (58).

Results also revealed that the intervention had no significant impact on outcomes of balance and mobility, with the notable exception of the CogTUG, and no impact on overall leisure participation. The absence of improvements on the Mini-BESTest and the single task version of the TUG could be due, in part, to the lack of alignment between the tasks involved in those assessments (largely related to locomotor and/or static skills) and the bicycling skills targeted in the intervention, to the short duration of the intervention, but also to the fact that participants involved in this study generally showed good balance and mobility skills at baseline (see participant characteristics in Table 3), with three participants displaying the maximal score on the Berg Balance Test and three displaying a TUG time within normative values (e.g., ≤10 s) (59, 60). As for the significant improvements observed on the CogTUG over the short duration of the training, it is reasonable to assume that they were induced by the multiple cognitive demands imposed by the practice of outdoor cycling during the training (i.e., awareness of the surrounding environment, planification of routes, mental flexibility to adapt to unexpected constraints, etc.). While the lack of improvement on the Nottingham Leisure Questionnaire may not be that surprising, considering the wide large variety of leisure activities covered by this questionnaire (from watching TV to practicing sports), it is worth mentioning that four participants continued to bike between the end of the intervention and the follow-up evaluation, which suggests that the intervention has the potential to lead to sustainable biking practice.

Last but not the least, this feasibility study intended to monitor adverse events. Two falls with minor sequels were reported, which while unfortunate was not entirely unexpected given the nature of the training. Safety was a major concern when designing this intervention and strategies were incorporated in the training to minimize falls, collisions and injuries. These include, amongst others, a safety checklist for therapist(s) to use, constant supervision and/or assistance of one or two therapists and the use of safety walking belts as needed. The choice of outdoor training environment also received special considerations and, for the present feasibility study, the parking lot (dedicated area delimited by cones, free of cars or unwanted obstacles) and surrounding area of the rehabilitation hospital was found to be a safe area yet sufficiently challenging to train the participants. This choice of environment may vary across different rehabilitation settings and would need to be determined on a case-by-case basis. To maximize safety during training but also for the independent practice of outdoor cycling, loss of balance and fall avoidance techniques were incorporated in Module 4. Participants were also screened in terms of their motor recovery, visual-perceptual abilities, cognitive function and balance capacity (see inclusion and exclusion criteria) to minimize the risk of falls. From a more global perspective, and with respect to the practice of cycling as an activity in everyday life, studies suggest that health benefits of cycling in the general population largely outweigh the risks associated with traffic accidents and inhaled air pollution (61) The increased accessibility to electrical bicycles to the general consumers, including older adults with or without stroke, may to some extent mitigate those benefits and pose new challenges in terms safety and approaches from clinicians.

This study has limitations, including a small sample size and a gender imbalance in recruited participants (4 males, 1 female), as well as conservative inclusion criteria in relation to the secondary objective of the study on feasibility, which limits the generalizability of the findings to the broader stroke population, particularly those with more severe sensorimotor and/or cognitive impairments who could potentially benefit from the activity with the appropriate adaptations (e.g., adapted bicycle, additional assistance and supervision). The sample size was limited by seasonal considerations in Canada and the availability of participants wanting to resume cycling during that period. The latter was likely due to a lack of awareness about the possibility of resuming cycling following stroke and the novelty of the intervention, which was then largely unknown from potential participants. Further studies involving larger sample sizes will be needed to support elements such as the intervention's efficacy, and to identifying who are the potential responders to this intervention. In addition, while the study shows that a three-week training was sufficient for participants to improve their confidence in their cycling skills and to achieve or even surpass their cycling goals, it cannot be ruled out that a longer intervention duration might have yielded even better outcomes.

## 5 Conclusion

In response to a need expressed by clinicians and their patients, as well as a significant gap in the literature, this study developed and tested a structured bicycle training program for stroke survivors with hemiparesis, which is now available to the community through this manuscript. The training protocol was found to be feasible, as demonstrated by participant adherence, progression, minimal occurrence of adverse events, and improvements in cycling-related outcomes. Further research could focus on determining the longer-term impact of outdoor cycling on health-related and functional outcomes, as well as identifying the minimum criteria for inclusion in such a training program following a stroke.

## Data Availability

The raw data supporting the conclusions of this article will be made available by the authors, without undue reservation.
