# Supplementary material for: Outdoor bicycle training following stroke: protocol development, feasibility testing and application guidelines
Source: Front Rehabil Sci. 2026 Mar 5;7:1661479. doi: 10.3389/fresc.2026.1661479 (PMC12999417; doi:10.3389/fresc.2026.1661479)
Supplement: Supplementary file 1 [file Table1.pdf]

Appendix A

Outdoor Cycling Safety Checklist		
**Bicycle**		
✓	**Task**	**Notes**
☐	Seat is horizontal	
☐	Height of the seat (straight leg when heel is on the pedal at 6 o'clock position)	
☐	Seat position (knee in line with pedal when it is at 3 o'clock position)	
☐	The back is as straight as possible	
☐	No shoulders elevated	
☐	Ball of the foot on the pedal	
☐	Wrist in neutral position	
☐	Palmar grip on the handlebar	

☐	Able to hold the brakes safely	
☐	Height of the handlebars	
	**Helmet**	
✓	**Task**	**Notes**
☐	Two finger widths above eyebrow	
☐	“V” shape under, and slightly in front of, the ears. Lock the slider if possible.	
☐	One to two fingers below chin strap	
☐	Ask participant to yawn; helmet should push downwards on head	
☐	Does the helmet rock back and forth more than two fingers above the eyebrow?	
☐	Does the helmet rock forward over the eyes?	
☐	Roll rubber band down to the buckle	

Appendix B

Bicycle Training Program - Module 1: Stationary Bicycle		
**Skills Targeted**	**Skills achievement checklist**	
		**Completed**
**Mounting the stationary bicycle**	Mounts stationary bicycle safely and independently	
**Initiating cycling**	Places pedals in starting position and initiate cycling	
**Cycling continuously with steady rhythm**	Pedals bike for 15 continuous revolutions to start then cycles for 1 minute without help	
**Shoulder checking**	Shoulder checks left and right without losing balance and cycling rhythm	
**Dismounting the stationary bike**	Dismounts stationary bicycle safely and independently	

Bicycle Training Program - Module 2: Bicycle with Training Wheels

Skills Targeted	Bicycling Skills checklist	
		Completed
**Walking with bicycle**	The participant will be able to walk with their bike, alternating between straight line walking and left/right turns around cones for 30m without any breaks	
**Getting on and off bicycle**	The participant will be able to mount and then dismount their bike safely and independently	
**Initiating pedaling and stopping without help**<br>**Maintaining Balance**	The participant will show a correct starting position (one pedal up, the other down) when initiating pedaling, will pedal in a straight line for 30m while maintaining balance, and then stop safely and independently	
**Straight line riding**	The participant will be able to cycle straight for 60m while staying within a 2m wide lane safely and independently	
**Stop quickly with control**	The participant will be able to come to a complete stop, quickly and with control, and then place one foot on the ground when prompted by the instructor 5 consecutive times	

**Making sharp turns**	The participant will be able to make four consecutive a 90° right hand turns, followed by another four consecutive 90° left hand turns	
**Making U-turns**	The participant will be able to make two consecutive wide right U-turns (approximately the width of a street) followed by two consecutive wide left U-turns	
**Steering to avoid obstacles**	The participant will be able to steer left and right to cycle around a straight line of 5 cones that are separated by 4 m (20 m)	
**Signaling**	The participant will be able to make all three signals (stop, left turn, right turn) and then perform the corresponding maneuver safely	
**Shoulder checking**	The participant will be able to look behind them over the left and right shoulder without losing balance or direction	
**Cycling on incline/decline**	The participant will be able to switch to a lower gear to cycle over a ramp, and then switch back to a higher gear	

Bicycle Training program - Module 3: Basic Bicycle <u>without</u> Training Wheels		
**Skills Targeted**	**Bicycling Skills checklist**	
		**Completed**
**Walking with bicycle**	The participant will be able to walk with their bike, alternating between straight line walking and left/right turns around cones for 30m without any breaks	
**Getting on and off bicycle**	The participant will be able to mount and then dismount their bike safely and independently	
**Initiating pedaling and stopping without help**<br><br>**Maintaining Balance**	The participant will show a correct starting position (one pedal up, the other down) when initiating pedaling, will pedal in a straight line for 30m while maintaining balance, and then stop safely and independently	
**Straight line riding**	The participant will be able to cycle straight for 60m while staying within a 2m wide lane safely and independently	

**Stop quickly with control**	The participant will be able to come to a complete stop, quickly and with control, and then place one foot on the ground when prompted by the instructor 5 consecutive times	
**Making sharp turns**	The participant will be able to make four consecutive a 90° right hand turns, followed by another four consecutive 90° left hand turns	
**Making U-turns**	The participant will be able to make two consecutive wide right U-turns (approximately the width of a street) followed by two consecutive wide left U-turns	
**Steering to avoid obstacles**	The participant will be able to steer left and right to cycle around a straight line of 5 cones that are separated by 4 m (20 m)	
**Signaling**	The participant will be able to make all three signals (stop, left turn, right turn) and then perform the corresponding maneuver safely	
**Shoulder checking**	The participant will be able to look behind them over the left and right shoulder without losing balance or direction	
**Cycling on incline/decline**	The participant will be able to switch to a lower gear to cycle over a ramp, and then switch back to a higher gear	

Bicycle Training program - Module 4: Advanced Bicycle Skills		
**Skills Targeted**	**Bicycling Skills checklist**	
		**Completed**
**Vary speed**	The participant will be able to increase and decrease their speed of biking while remaining in control. Done on command, without anticipatory knowledge of sequence of actions.	
**Practice loss of balance**	The participant will be able to drop bike and dismount quickly to avoid a fall when stationary.	
**Stall and resume biking**	The participant will be able to come to a stop, stall the bike for 3 seconds and resume biking to a comfortable speed	
**Passing another cyclist and being passed**	The participant will signal to the cyclist in front using bell or voice and pass safely on the left. The participant will remain at a constant speed and in a straight-line while being passed by another cyclist.	
**Ride over obstacles**	The participant will ride over a 3 2x4 boards, separated by 3m.	

	Options: 1) Roll over, 2) Front wheel lift off 3) Bunny hops	
**Drops: Ride down/off a curb**	The participant will ride off a standard sidewalk curb and maintain control upon completion.	
**Narrow biking**	-Bike in a narrow path (0.75m) for 15m and maintain speed to avoid handlebar contact with surroundings.<br><br>-Ride 1m at a width (0.10)<br><br>-Ride through 3 sets of cones slalom track, between with each pair of cones at a width of (0.1m) separated by 3m.	
**Biking while standing**	-In a flat area: transition from seated to standing, and remain standing for 10m, and return to a seated position with control.<br><br>-Bike on an inclined surface, transition from seated to standing, remain standing for 10m and return to a seated position, all with control.	
**Biking single hand**	Bike in a straight line for 20m only using left/right hand.	
**Single leg pedaling**	Bike for 20m using only one foot (opposite foot remains on the pedal).	

**Narrow slalom**	The participant will be able to steer left and right to cycle around a straight line of 5 cones that are separated by 2 m.	
**Narrow 360 degree turns when surrounded by obstacles**	Complete left and right turns within a 3m radius, avoiding obstacles on the outer aspect of the turn.	
**Predictable obstacle course**	60m obstacle course where the course is explained, the participant can observe a demo and complete a walk through prior to.	
**Unpredictable obstacle course**	60m obstacle course, where route changes as participant completes the route.	
**Application of all skills learned, in an uncontrolled setting**	Safely complete a 10km bike path loop integrating all skills learned.	

Appendix C

Participant	Week 0	Week 3	Week 6	Week 9
Mini-BESTest				
1	19	20	22	20
2	22	22	21	21
3	24	21	21	23
4	26	25	27	28
5	23	24	27	27
TUG				
1	9,8	10,5	7,7	8,73
2	13,5	12,63	10,66	10,55
3	9,51	10,15	9,55	7,95
4	6,5	6,58	7,25	6,96
5	8,98	8,69	8,5	7,61
Cognitive TUG				
1	12,1	10,6	8,09	10,03
2	15,63	18,3	13,35	14,25
3	12,48	13,36	11,2	8,21
4	8,12	9,1	7,6	6,63
5	9,25	8,9	8,38	8,19
Nottingham Leisure Questionnaire				
1		32	28	23
2		26	30	31
3		29	29	30
4		31	29	29
5		34	35	35
